# Transgenic soybean expressing a thermostable phytase as substitution for feed additive phytase

**DOI:** 10.1038/s41598-019-51033-y

**Published:** 2019-10-07

**Authors:** Yu Zhao, Lixia Zhu, Chaoyang Lin, Zhicheng Shen, Chao Xu

**Affiliations:** State Key Laboratory of Rice Biology, Institute of Insect Sciences, College of Agriculture and Biotechnology, Zhejiang University, Hangzhou, China

**Keywords:** Molecular engineering in plants, Hydrolases

## Abstract

Phytase is one of the most effective feed additives to increase the availability of phosphorus and minerals by catalyzing the hydrolysis of phytic acid. A modified *appA* gene (*mappA*) was transformed into soybean (*Glycine max*) under the control of a seed-specific promoter from common bean (*Phaselous vulgaris*). The soybean recombinant phytase showed optimal activity at pH 4.5 and 70 °C. A slight increase in enzyme activity occurred when the recombinant enzyme was pre-incubated with *n*-hexane. In addition, the phytase activity from our transgenic soybean does not reduce even after 2 hours of extraction with *n*-hexane at 55~65 °C. In conclusion, the oil extraction process using *n*-hexane does not inactivate the phytase expressed in the mAppA transgenic soybean, and the meal derived from the transgenic soybean processing can be used as feed supplement to livestock.

## Introduction

Phytate (*myo*-inositol 1, 2, 3, 4, 5, 6-hexakisphosphate) is the major form of phosphorus found in plant-based feeds such as cereals, legumes and oilseed crops^1–3^. Phytate phosphorus is not readily available to monogastrics^4,5^, thus phosphate supplementation is required for optimal animal growth^6^. However, release of undigested phytate through manure can lead to eutrophication in areas of intensive livestock production^7,8^. Phytate can chelate important cations, such as iron, zinc, magnesium, manganese, copper and calcium, and may also bind protein to form phytate-cation-protein complexes that lower the bioavailability of minerals and amino acids in feed^9–11^.

Phytase (*myo*-inositol hexakisphosphate phosphohydrolase) hydrolyzes phytate to less-phosphorylated *myo*-inositol derivates and inorganic phosphate. Exogenous phytase is supplemented in the diet of monogastrics because of the absent or limited phytase activity in their digestive tract^12^. In this matter, phytase can improve the dietary availability of phosphorus and minerals and has emerged as one of the most effective feed additives^10,11^. Phytases are broadly found in plants, animals and microorganisms^8^. Microbial phytases have been employed in feed industry and are commercially produced by submerged fermentation techniques (SmF)^13,14^. Currently, the production of phytase by solid-state fermentation techniques (SSF) has gained a fresh attention from researchers^14–18^. As compared to SmF, SSF possesses higher product concentration, better product recovery, lower catabolic repression and lower capital cost^19,20^. However, SSF is still under research due large scale production and enzyme purification limitations. Developing phytase transgenic crops represents another effective way to increase phosphorus availability and eliminate the need for phytase supplementation.

Soybean (*Glycine max*) is widely harvested worldwide to be used as food or raw material for oil and protein production. In feed industry, soybean meal is obtained after oil extraction as forage to livestock due its abundant protein content^21,22^. Phytase has been reported to be expressed in transgenic soybean previously. Some of the transgenic soybean was generated to express phytase to reduce phytate content of soybean itself ^23–26^, while other transgenic soybean was created to provide an alternative to phytase supplementation^26,27^. Of note, a soybean line (CAPPA) expressing an *Escherichia coli appA* gene driven by the soybean lectin promoter exhibited a high level of phytase expression (>1000 units g^−1^ seed)^26^, making its meal as a suitable replacement of commercial fungal phytases. The same gene was previously transformed into *Arabidopsis thaliana* and the transgenic seed phytase exhibited an optimum temperature of 50 °C and remained <5% of activity after incubation at 70 °C for 20 min^28^. It is highly desirable to develop transgenic soybean expressing a thermostable phytase to accommodate soybean processing procedures which often endure high temperature. In the present study, we generated a transgenic soybean expressing a modified thermostable phytase mAppA^29^. Transgenic soybean retained its high phytase activity after oil extraction by *n*-hexane. Soybean meal derived from mAppA transgenic soybean seeds can be used as sources of phytase as alternative to manual phytase addition after oil extraction.

## Results

### Generation of soybean events expressing mAppA

**Figure 1:**
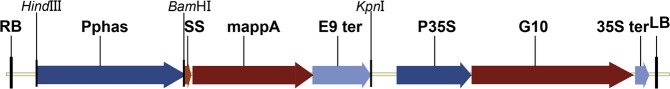
Diagram of the T-DNA used for soybean transformation. RB and LB, right border and left border of T-DNA respectively; Pphas, promoter of the *β-phaseolin* gene; SS, signal peptide of the 2S2 seed storage protein gene of *A. thaliana*; mappA, the modified thermostable phytase gene; E9 ter, the rbcSE9 terminator of *Pisum sativum*; P35S, CaMV 35S promoter; G10, 5-enolpyruvylshikimate-3-phosphate synthase (EPSPS) gene; 35S ter, CaMV 35S terminator.

To create the transgenic soybean events expressing the mAppA, we constructed a T-DNA consisting of the phytase gene expression and the glyphosate resistance cassettes (Fig. 1). The *mappA* gene expression cassette is composed by the promoter of the common bean storage protein β-phaseolin, the codon-optimized synthetic *mappA* gene and a terminator. The glyphosate resistance cassette is used as the selection marker for soybean transformation. The T-DNA was transformed into the elite soybean cultivar ‘Wandou-28’ by *Agrobacterium*-mediated transformation.

### Expression of mAppA in seeds of transgenic soybean

Forty transgenic lines were obtained and T1 seed batches were analyzed for their phytase content. The expression levels ranged from 0.29 to 350 U/g seed. Four events with the highest phytase activity were selected for further analysis.

**Figure 2:**
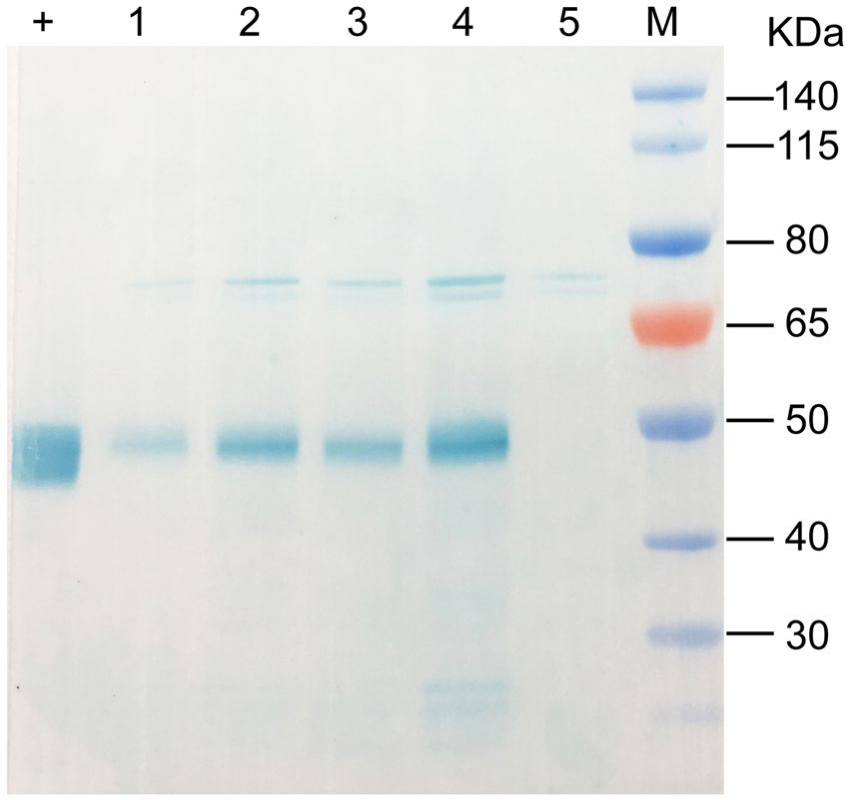
Western blot analysis of the transgenic soybean expressing phytase. Lane M: molecular weight marker; +: mAppA expressed in *E.coli* (positive control); lanes 1–4: samples from different transgenic soybean lines; lane 5: sample from non-transgenic soybean (negative control).

Crude protein extracts were prepared from seed batches of transgenic lines. The extracts were analyzed by western blot analysis using antisera against mAppA. A band of about 45 kDa was detected among different transgenic lines (Fig. 2, detailed information shown in supplementary Figure S1) showing phytase expression and its molecular weight (45.3 kDa).

Event Phs-39 expresses the highest level of phytase as suggested by activity assay. We investigated the major agronomic traits of this line. Plant height and pods per plant of Phs-39 were similar to non-transgenic soybean (Table 1). The germination rate of Phs-39 line was not reduced (data not shown). However, we observed a 4% reduction in 100-grain weight in Phs-39 compared to the non-transgenic crop (Table 1). In addition, transgenic rice seeds expressing cellulase^30^ and lipase^31^ also presented reduction in seed weight. The high level expression of xenogeneic enzymes likely has cost the seed weight.

**Table 1:** Major agronomic traits of the transgenic and non-transgenic soybean plants (Wandou 28) grown under field conditions.

	Plant height (cm)	Pods per plant	100-grain weight (g)
Wandou 28	28.4 ± 2.5	24.5 ± 6.5	27.2 ± 0.6
PHS-39	27.9 ± 2.3	25.6 ± 5.6	26.1 ± 0.2*

Values are mean ± SD.

*P < 0.05 by the Student’s *t* test.

### Characterization of mAppA expressed in soybean seeds

**Figure 3:**
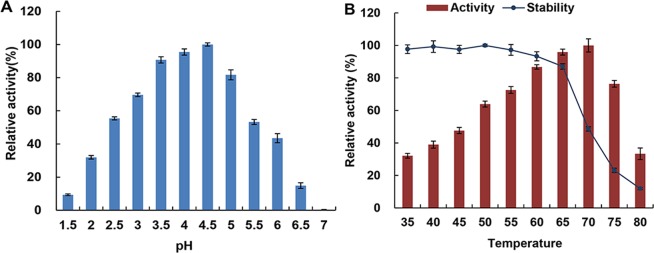
Effect of pH and temperature on the hydrolytic activity of phytase. (**A**) Phytase activity at different pHs. (**B**) Phytase activity at different temperatures. Data are showed as the mean ± SD (n = 3).

The phytase from transgenic soybean had a pH optimum of 4.5 (Fig. 3A). Phytase exhibited more than 80% of its maximal activity at pHs between of 3.5 to 5.0. pHs above 6.5 or at pH 1.5 exerted an inhibitory effect on the enzyme.

The phytase from transgenic soybean showed a temperature optimum of 70 °C (Fig. 3B). The enzymatic activity gradually increased with the temperature up to 70 °C, while the activity decreased above 70 °C. Temperature stability assay showed the enzyme remained stable below 65 °C, and its stability declined at higher temperatures (Fig. 3B).

### Kinetic parameters of the phytase

The kinetic properties of the phytase were determined by incubation with different concentrations of sodium phytate: 0.0125 mM, 0.025 mM, 0.05 mM, 0.1 mM, 0.2 mM, 0.4 mM, 0.8 mM, 1.6 mM, 3.2 mM or 6.4 mM. Our results show that the average *Km* value for the phytase extracted from *E. Coli* (purified 6 × His-fused recombinant protein) or the transgenic soybean (without purification) was 98.6 ± 19.8 μM and 103 ± 35.2 μM, respectively. Thus, the enzymatic kinetics of the protein expressed in the soybean is similar to that expressed in the bacteria.

### Effect of metal ions on phytase activity

The effect of different metal ions on phytase activity was assessed by incubating the enzyme with different metal ions (K^+^, Mn^2+^, Mg^2+^, Cu^2+^, Zn^2+^, Ca^2+^ or Co^2+^) at different molar concentrations (1 mM or 5 mM) for 1 h at room temperature. Our results indicated that phytase activity was not significantly affected by most of ions tested (Table 2). The phytase activity was inhibited by Zn^2+^ and Cu^2+^ at 1 mM or 5 mM (Table 2).

**Table 2:** Effects of metal ions on the enzymatic activity of the recombinant phytase.

Metal ions and Control	Relative activity (%)	
	1 mM	5 mM
No addition	100.0 ± 2.9	100.0 ± 4.4
KCl	96.4 ± 2.0	91.3 ± 2.0
MnCl_2_	96.6 ± 3.2	91.1 ± 3.5
MgCl_2_	97.0 ± 2.2	103.4 ± 3.4
CuCl_2_	89.6 ± 0.2	49.9 ± 3.0
ZnSO_4_	77.5 ± 1.4	52.3 ± 2.4
CaCl_2_	100.8 ± 4.2	95.6 ± 5.4
CoCl_2_	101.2 ± 1.9	99.3 ± 5.1

### Effect of organic solvents on phytase activity

**Figure 4:**
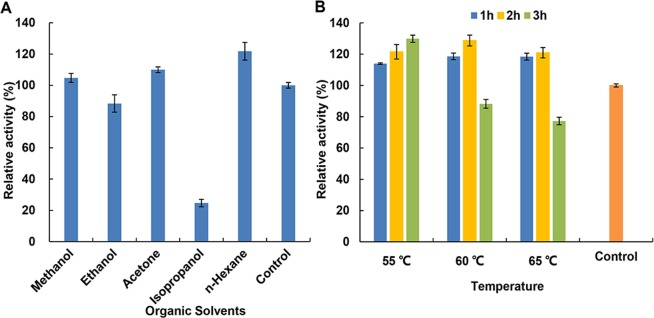
Effects of organic solvents on the enzymatic activity of the phytase produced in soybean and the tolerance of recombinant phytase in *n*-hexane. (**A**) Effects of organic solvents on the activity of recombinant phytase. Control, sample without being incubated with any solvents. (**B**) Effects of *n*-hexane-extraction on recombinant phytase activity. Control, sample without being extracted with *n*-hexane. The residual activity was determined using the standard assay method, and the activity of the untreated control was set to 100%. Data are showed as the mean ± SD (n = 3).

Extraction by organic solvent is the most common and efficient method for oil production. However, different polarity of organic solvents may modify the activity of phytase. We investigated whether the activity of phytase can be modified after being pretreated with different organic solvents for 1 h. The phytase activity was significantly inhibited by isopropanol (75.3% reduction) and slightly inhibited by ethanol (11.6% reduction). Of note, increased enzyme activity was observed after treatment with methanol (4.7% increase), acetone (10.0% increase) or *n*-hexane (21.8% increase) (Fig. 4A).

### Effect of *n*-hexane-extraction on phytase activity

To further confirm the effects of *n*-hexane on phytase activity, this organic solvent was used to extract oil from the transgenic soybean at different temperatures and time points. High phytase activity, ranging from 114.0% to 128.7%, was observed when the extraction occurred within two hours (Fig. 4B). Thus, the coproduct of soybean oil production, the soybean meal, has the potential to be used as feed additive.

## Discussion

Phytase is an important additive to monogastrics diet, due its ability to increase phosphorus accessibility and to reduce excessive phosphorus accumulation in manure. Phytase supplementation can also reduce rock phosphorus use and is conducive to resource preservation^32^. Field studies showed that the addition of 500 to 1,000 units kg^−1^ of phytase to feed diet is analogous to 1% dicalcium phosphate or 0.18% phosphorus supplementation^5^. In the present study, we report the presence of 350 units of phytase per gram of seed on our mAppA soybean, suggesting that only 2~3 kg of the transgenic soybean may be required to supplement 1 metric ton of feed. Our mAppA transgenic soybean can be used as a natural additive to feed without downstream processing.

Solvent extraction is the most common and efficient method for oil production. Therein, commercial hexane (45–90% *n*-hexane) is the most frequently used solvent in the industry^33,34^. We evaluated the effect of five organic solvents on the catalytic properties of phytase. It was noticed that phytase treated with *n*-hexane showed higher activity than control. Similar phenomenon has been observed when extracellular phytase from *Aspergillus niger* van Teighem was incubated with hexane (10% v/v)^35^. These results suggest that *n*-hexane has no deleterious effect on active site residues of phytase.

The process to extract oil from soybean using industrial settings, takes about 25 min (contact time)^36^, and most extractions occur at 60 °C to provide a safety margin below the solvent boiling range^36^. In our protocol, we found a slight increase in phytase activity after extraction using *n-*hexane between 55 to 65 °C along 2 hours, indicating this enzyme can sustain the oil extraction process at high temperatures. This is partially due the thermostable characteristic of the phytase expressed in our transgenic soybean. Soybean meal is the by-product of oil extraction from soy and it is the most important protein source used to feed livestock. Soybean meal represents two-thirds of the total world output of protein feedstuffs, including other major oil and fish meals^21^. Thus, the soybean meal derived from the mAppA transgenic soybean showed sufficient enzyme activity to serve as a feed additive.

In conclusion, the mAppA transgenic soybean can be used as a source phytase supplementation to feed livestock even after oil extraction. Its application in feed industry can decrease enzyme purification steps and production costs.

## Methods

### Vector construction

pCambia1300 (Cambia, Canberra, Australia) was digested with *Xho*I to substitute the hygromycin-resistant gene sequence with a glyphosate-tolerant *G10* gene sequence^37^. Thus, the open reading frame was brought under control of the CaMV 35S promoter and the CaMV 35S terminator. The modified vector was named as p1300-G10, and was further used to clone the phytase expression cassette.

The phytase gene *mappA* with the N-terminal signal sequence of the seed storage protein 2S2^38^ and the rbcSE9 terminator of *Pisum sativum* (GenBank Accession No. **X00806.1**) was codon-optimized and synthesized by Sangon Biotech (Shanghai) Corporation Limited. A *Bam*HI site was introduced at the 5′ end, and a *Kpn*I site was added to the 3′ end of the synthetic sequence. Genomic DNA of *Phaselous vulgaris* cv. Velour was isolated^39^ and used as a template in a PCR reaction. The *β-phaseolin* promoter (GenBank Accession No. **J01263.1**) was amplified using a forward primer (5′-AAGCTTATTGTACTCCCAGTATC-3′, the *Hin*dIII restriction site is underlined) and a reverse primer (5′-GGATCCGAAAGAAGTGAGTGATATTAG -3′, the *Bam*HI restriction site is underlined). The *β-phaseolin* promoter fragment was digested with *Hin*dIII and *Bam*HI, the synthetic gene fragment was digested with *Bam*HI and *Kpn*I, and the vector p1300-G10 was predigested with *Hin*dIII and *Kpn*I. The promoter, gene fragment, and the vector were further ligated to generate the p1300-mappA-G10 plasmid. This construct includes two expression cassettes and the glyphosate resistance and the phytase expression cassettes.

### *Agrobacterium*-mediated transformation

p1300-mappA-G10 plasmids were transformed into *Agrobacterium tumefaciens* EHA105 by electroporation. Elite soybean cultivar ‘Wandou-28’ [Glycine max (L.) Merrill] was transformed using an *Agrobacterium*-mediated transformation method^40^. Glyphosate (Sigma-Aldrich) was used as selection agent for transgenic soybean plants from cotyledon explants.

### Expression and purification of his-tagged recombinant protein

Using ClonExpress®II One Step Cloning Kit (Vazyme Biotech Co., Ltd.), a full-length ORF encoding the *mappA* gene was cloned into the pET28a (Merck Millipore, Darmstadt, Germany) vector. The recombinant vector (pET28a-mappA) was transformed into *Trans*etta (DE3) (TransGen Biotech). Expression mAppA and purification of 6 × His-fused recombinant protein were carried out according to the manufacturer’s protocol (Qiagen, Hilden, Germany). The purified 6 × His-fused protein was dialyzed overnight and analyzed by SDS-PAGE. Positive samples were then used as an immunogen for the preparation of polyclonal rat antisera. The purified 6 × His-fused protein was quantified against bovine serum albumin standard according to the recommendations of the manufacturer of the BioRad protein assay.

### Western blot analysis

Seed samples weighting 0.1 g were milled, homogenized in 1 mL PBS buffer and centrifuged at 12396 g for 10 min at 4 °C. The supernatant was collected and the expression of mAppA in the transgenic soybean was verified by Western Blot analysis. The proteins were separated by SDS-PAGE and then transferred to a PVDF membrane (Pall, Ann Arbor, MI, USA). Primary rabbit anti-mAppA polyclonal antibodies were prepared from rabbits immunized with *E*. *coli* expressing mAppA protein. The anti-mAppA antibody was then detected by horseradish peroxidase-conjugated goat anti-rabbit IgG (Sigma-Aldrich) as the secondary antibody. TMB stabilized substrate solution for Horseradish Peroxidase (Promega catalog number W4121) was used for detection.

### Phytase activity assay

Phytase activity was detected in protein extracts from mature seeds. Samples weighting 0.1 g were pulverized in a mortar and protein extracts were homogenized in 10 mL 100 mM sodium acetate buffer, pH 5.0. The resulting mixture was clarified by centrifugation (15938 g, 10 min) and the supernatant was collected and assayed for phytase activity by the ferrous sulfate-molybdenum blue method^41^ with slight modification: 150 µL aliquots of the protein extracts were incubated with 600 µL of 6.25 mM sodium phytate in 100 mM sodium acetate buffer pH 5.0 for 30 min at 37 °C. The reaction was stopped by adding 750 µL of a 5% (w/v) trichloroacetic acid solution. Color reagent was added to the sample solution and the production of phosphomolybdate was measured by spectrophotometer at 700 nm. One unit (U) was defined as the amount of enzyme required to release 1 μmol orthophosphate from phytic acid per minute under assay conditions.

### Effect of pH on phytase activity

The pH optimum for phytase activity was determined using different solutions ranging from pH 1.5 to 7 at 37 °C: 0.2 M glycine-HCl (pH 1.5–3.5), 0.2 M sodium acetate – acetic acid (pH 4–5.5), 0.2 M MES (pH 6–6.5), and 0.2 M Tris-HCl (pH 7).

### Effect of temperature on phytase activity and stability

The temperature optimum for phytase activity was measured using the standard assay at different temperatures from 35 to 80 °C. Temperature stability of phytase was determined by incubation of enzyme in 0.2 M sodium acetate buffer for 30 min at temperatures from 35 to 80 °C and assaying the residual phytase activity in accordance with the standard assay.

### Effect of organic solvents and metal ions on phytase activity

To determine the effect of organic solvents on phytase activity, 45 µL of methanol, ethanol, acetone, isopropanol, *n*-hexane or 100 mM sodium acetate buffer, pH 5.0 (as the control), was added individually to 105 µL of properly diluted crude enzyme extracts and mixed by vortexing. After incubated at room temperature for 1 h, the enzyme activity of the mixtures was measured using the standard assay. The activity of the enzyme without organic solvents was used as control and set at 100%. All measurements were repeated three times.

To investigate the effect of different metal ions on phytase activity, the enzyme was pre-incubated with 1 mM or 5 mM of KCl, MnCl_2_, MgCl_2_, CuCl_2_, ZnSO_4_, CaCl_2_ or CoCl_2_ at room temperature for 1 h. Enzyme samples without metal ions treated were used as control. The residual activity of the enzyme was measured using the standard assay.

### Effect of *n*-hexane-extraction on phytase activity

Mature seeds from the mAppA transgenic line were pulverized to permit passage through a 0.38 mm sieve^36^. The extraction mixture consisted of 1 g of collected soybean flour^42^ and 5 mL of *n*-hexane (97%, Sinopharm Chemical Reagent Co., Ltd). The ratio of solvent to beans was 5:1^43^. Experiments were performed at 55, 60 or 65 °C^44,45^, with extraction time limit set to 1, 2 or 3 hs at each temperature point. All the extractions were carried out in triplicate. At the end of the contact time, the supernatants were discarded by centrifugation (15938 g, 10 min) and traces of solvent remaining were removed by evaporation. The resulting precipitation was homogenized in 10 mL of 0.2 M sodium acetate buffer (pH 5.0) for phytase extraction. The phytase in the control sample (1 g of soybean flour without *n*-hexane extraction) was extracted simultaneously with 10 mL of 0.2 M sodium acetate buffer (pH 5.0). After centrifugation (15938 g, 10 min), the supernatants were collected and assayed for phytase activity using the standard method. The phytase activity per gram for both non *n*-hexane-extracted and *n*-hexane extracted samples were calculated based on original pulverized soybean weight.

### Characterization of the kinetic parameters of phytase

The purified 6 × His-fused phytase and unpurified soybean recombinant phytase were diluted with 50 mM sodium acetate buffer, pH 4.5, to a final concentration of 0.2 U/mL. Phytase activity was determined at 37 °C using sodium phytate as substrate at 10 different concentrations ranging from 12.5 μM to 6.4 mM in a 5 min assay (n = 3). The software Origin 8.0 was used for nonlinear regression analysis of the enzyme kinetics data.

### Evaluation of major agronomic traits of the mAppA transgenic soybean

Data about plant height, pods per plant and the 100-grain weight were collected at the Experimental Station of Zhejiang University in Sanya City (Hainan Province) during December 2018 to March 2019. All accessions were planted in three-row plots in 500-cm-long rows with spacing of 50 cm between rows. The experiment was design as a randomized complete block with three replications. All plots of transgenic and non-transgenic soybean (Wandou 28) were uniformly managed to control water, fertilizer, disease and pest. At maturity, major agronomic characteristics, such as plant height, pods per plant, and the 100-grain weight, were evaluated.

## Supplementary information

Figure S1
