# Supplementary material for: Transgenic soybean expressing a thermostable phytase as substitution for feed additive phytase
Source: Sci Rep. 2019 Oct 7;9:14390. doi: 10.1038/s41598-019-51033-y (PMC6779883; doi:10.1038/s41598-019-51033-y)
Supplement: Supplementary file 1 — Figure S1 [file 41598_2019_51033_MOESM1_ESM.pdf]

Transgenic soybean expressing a thermostable phytase as substitution for feed additive phytase

Yu Zhao[a], Lixia Zhu[a], Chaoyang Lin[a], Zhicheng Shen[a] and Chao Xu[a]*

[a]State Key Laboratory of Rice Biology, Institute of Insect Sciences, College of Agriculture and Biotechnology, Zhejiang University, Hangzhou, China

*Correspondence and requests for materials should be addressed to C. Xu. (email: zjuxuchao@163.com)

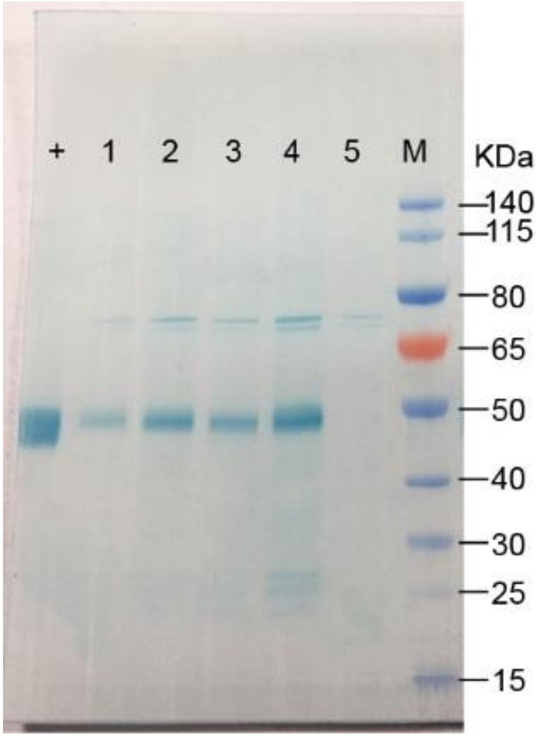

Figure S1. Western blot analysis of the transgenic soybean expressing phytase. Lane M: molecular weight marker; +: mAppA expressed in *E.coli* (positive control); lanes 1-4: samples from different transgenic soybean lines; lane 5: sample from non-transgenic soybean (negative control).
